# Serum folate levels and risk of metabolic dysfunction-associated steatotic liver disease: results from a cross-sectional study and Mendelian randomization analysis

**DOI:** 10.3389/fnut.2024.1437183

**Published:** 2024-09-04

**Authors:** Yalan Chen, Jie Gao, Xibin Wang, Hong Lu, Ya Zheng, Qian Ren

**Affiliations:** ^1^The First School of Clinical Medicine, Lanzhou University, Lanzhou, Gansu Province, China; ^2^Department of Gastroenterology, The First Hospital of Lanzhou University, Lanzhou, Gansu Province, China; ^3^Gansu Province Clinical Research Center for Digestive Diseases, Lanzhou University, Lanzhou, Gansu Province, China

**Keywords:** folate, Mendelian randomization, NHANES, MASLD, cross-sectional study

## Abstract

**Background:**

Evidence from observational studies on the association between folate and metabolic dysfunction-associated steatotic liver disease (MASLD) is conflicting.

**Aims:**

This study aimed to investigate the association between serum folate concentration and MASLD and further assess the causal relationship using Mendelian randomization (MR) analysis.

**Methods:**

To investigate the causal relationship between serum folate and MASLD, we conducted a cross-sectional study that selected 1,117 participants from the 2017–2020 National Health and Nutrition Examination Survey (NHANES). The association between serum folate level and the risk of MASLD was evaluated under a multivariate logistic regression model. In addition, we conducted a two-sample MR study using genetic data from a large genome-wide association study (GWAS) to compare serum folate level (37,465 individuals) and MASLD (primary analysis: 8,434 cases/770,180 controls; Secondary analysis:1,483 cases/17,781 controls) were performed to infer causal relationships between them. Inverse variance weighted (IVW) was used as the primary method of MR Analysis.

**Results:**

The results from the NHANES database showed that Tertile 3 group (Tertile 3: ≥ 48.6 nmol/L) had a significantly lower risk (OR = 0.58, 95% CI: 0.38–0.88, *p* = 0.010) of MASLD than Tertile 1 group (Tertile 1: < 22.3 nmol/L) after complete adjustments. However, in the IVW of MR analysis, there was no causal relationship between serum folate level and MASLD risk in the primary analysis (OR = 0.75, 95% CI: 0.55–1.02, *p* = 0.065) and secondary analysis (OR = 0.83, 95% CI: 0.39–1.74, *p* = 0.618).

**Conclusion:**

In observational analyses, we observed an inverse association between higher serum folate concentrations and a reduced risk of MASLD. Our MR study generated similar results, but the association failed to reach the significance threshold of *p* < 0.05, suggesting that our MR study does not support a causal relationship between serum folate levels and MASLD risk. Additional research involving a larger number of cases would contribute to enhancing the confirmation of our preliminary findings.

## 1 Introduction

Metabolic dysfunction-associated steatotic liver disease (MASLD) is the leading cause of end-stage liver disease globally, with an estimated global prevalence of about 29.8% (1). MASLD poses significant threats to human health and imposes substantial economic burdens on society. MASLD is characterized by steatosis affecting more than 5% of liver cells and is unrelated to heavy alcohol consumption, lipid-causing drugs, or genetic disorders (2). MASLD includes various chronic liver conditions like simple hepatic steatosis, non-alcoholic hepatitis (NASH), and liver fibrosis (3, 4). Although the etiology of MASLD remains unclear, multiple factors such as oxidative stress, gut microbes, lipid metabolism, genetic susceptibility, insulin resistance, and some nutritional and lifestyle factors have been shown to work together to drive the development of MASLD (5, 6). However, there is currently no globally approved drug therapy for MASLD treatment (7). Therefore, it is particularly important to explore new intervention mechanisms, therapeutic agents, and targets for MASLD.

Folate, a water-soluble vitamin vital for one-carbon metabolism and methylation reactions, has been implicated in processes such as oxidative stress, liver fat metabolism, and chronic liver inflammation, all recognized risk factors for MASLD (8). Animal studies have demonstrated that folate supplementation reduces hepatic steatosis (9, 10). Previous epidemiological analyses have indicated an inverse relationship between higher serum total folate concentrations and the prevalence of MASLD and liver fibrosis (11). A recent study employed meta-analysis and MR analysis to illustrate an inverse association between serum folate and MASLD risk (12). However, past evidence has been inconsistent at times. A meta-analysis of eight studies published up to 2015 found no significant difference in folate concentrations between patients with and without MASLD (standardized mean difference: −0.26 nmol/L; 95% CI: −0.69 to 0.17) (13). Two recent cross-sectional studies among MASLD-related populations in the United States reported no observed association between serum or dietary folate and MASLD (14, 15). Although the main topic of their study was not serum folate, it does suggest that the association between serum folate and MASLD remains controversial. Therefore, further investigation into the association between serum folate levels and MASLD is warranted.

To explore the association between serum folate concentration and the risk of MASLD, we conducted an observational study utilizing data from the National Health and Nutrition Examination Survey (NHANES) database for the United States population (16). Additionally, we employed MR analysis. This epidemiological approach leverages genetic instrumental variables (IVs) to infer causality and evaluate the causal link between circulating folate levels and the risk of MASLD. MR circumvents potential confounding effects from environmental risk factors and disease progression, as an individual’s genotype is fixed at conception and remains unaffected by environmental and lifestyle factors (17). Consequently, genetic variants are recommended as IVs to mitigate reverse causality and other confounding factors. The present study systematically examined the genetic causal association between folate and MASLD risk, employing case–control and two-sample MR analyses. The findings of this study offer novel insights into clinical prevention and treatment strategies.

## 2 Materials and methods

### 2.1 Cross-sectional study

#### 2.1.1 Study design and participants

National Health and Nutrition Examination Survey, a continuous national survey program administered by the National Center for Health Statistics (NCHS), aims to evaluate the health and nutritional standing of adults and children in the United States. The selection of participants followed the stratified, multistage sampling design employed by NHANES. Participants in this study underwent interviews covering demographics, socioeconomic factors, dietary habits, and health status. All participants provided their written informed consent prior to participation.

**Figure 1:**
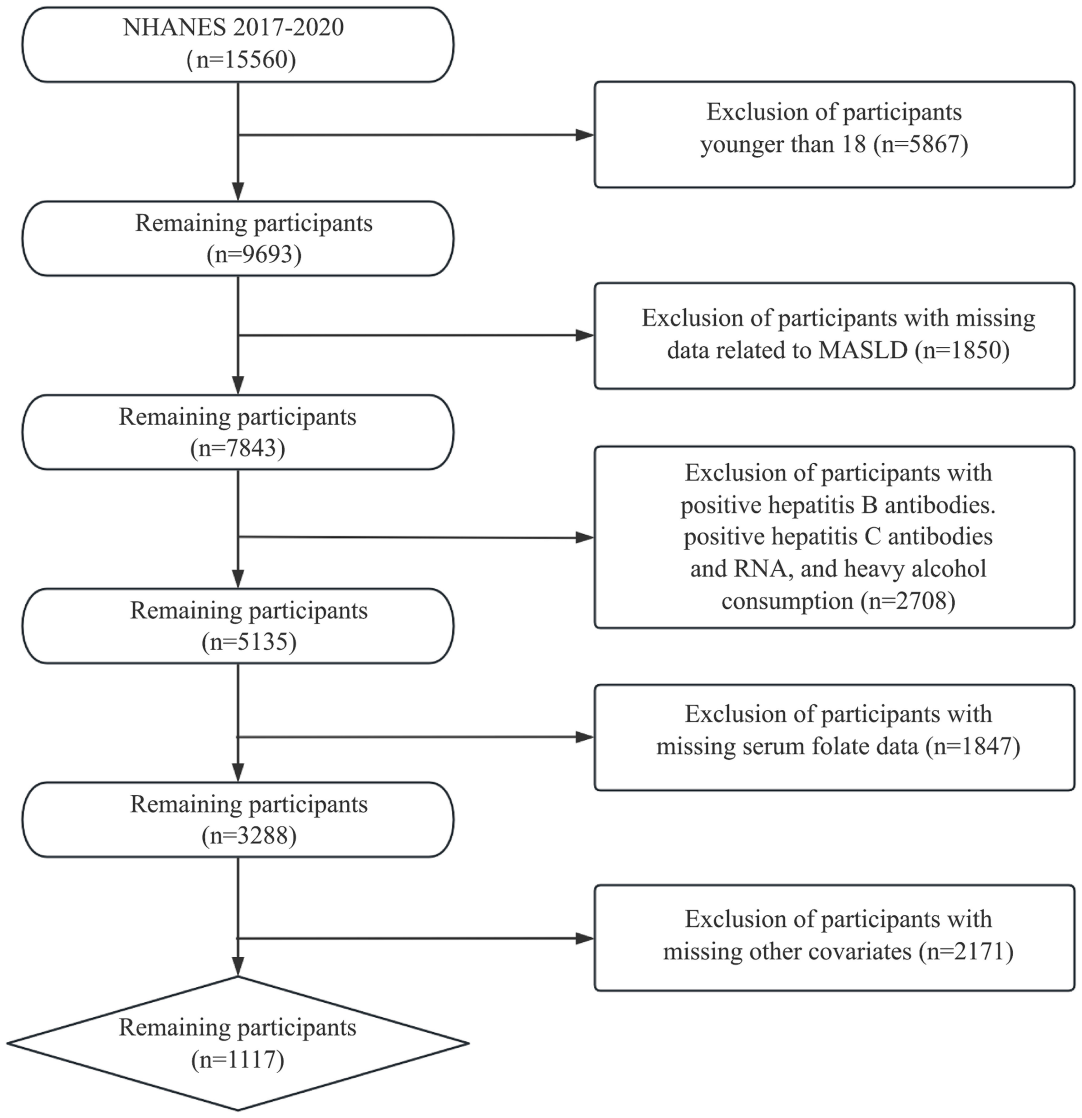
Flow chart for inclusion and exclusion of participants.

We utilized samples from the 2017–2020 NHANES survey with MASLD diagnoses to investigate the association between serum folate concentration and MASLD. Among the initial cohort of 15,560 participants, the following exclusion criteria were applied: (1) age < 18 years; (2) hepatitis B antibody, hepatitis C antibody, or hepatitis C virus RNA positive; (3) excessive alcohol drinkers (male >30 g/day, female >20 g/day); (4) participants with incomplete data on fatty liver index or serum folate; and (5) missing data for other relevant covariates. Consequently, the final sample comprised 1,117 participants. Figure 1 depicts the process of participant inclusion and exclusion.

#### 2.1.2 Measurement of serum folate

In the NHANES, serum folate concentrations were quantified using isotope dilution high-performance liquid chromatography–tandem mass spectrometry (LC–MS/MS) (18). The serum samples were initially mixed with an ammonium format buffer and an internal standard mixture. Next, automated solid-phase extraction (SPE) using a 96-well phenyl SPE plate was employed for sample extraction and purification. Within 6 min, the folate form was isolated using an isocratic mobile phase condition and analyzed using LC–MS/MS.

#### 2.1.3 Measurement of MASLD

In this study, the diagnosis of MASLD in adults was determined by measuring the controlled attenuation parameter (CAP) using vibration-controlled transient elastography (VCTE). Hepatic steatosis was assessed based on a CAP threshold of ≥248 dB/m; values exceeding this threshold indicated the presence of hepatic steatosis (19). MASLD is defined as hepatic steatosis combined with metabolic dysfunction, without other causes of hepatic steatosis. Metabolic dysfunction is diagnosed if at least one of the following criteria is met: (1) Body Mass Index (BMI) ≥ 25 kg/m^2^ or waist circumference > 94 cm for men and > 80 cm for women (adjusted for race); (2) Fasting blood glucose ≥5.6 mmol/L, 2-h postprandial blood glucose ≥7.8 mmol/L, HbA1c ≥ 5.7%, or a diagnosis of type 2 diabetes or current treatment for type 2 diabetes; (3) Blood pressure ≥ 130/85 mmHg or use of antihypertensive medication; (4) Plasma triglycerides(TG) ≥ 1.7 mmol/L or treatment for elevated blood lipids; and (5) Plasma high-density lipoprotein cholesterol(HDL-C) ≤ 1.0 mmol/L for male or ≤ 1.3 mmol/L for female, or treatment for elevated blood lipids.

#### 2.1.4 Statistical analysis

R version 4.3.2 was used for all analyses. Categorical variables were described using counts and proportions, while continuous data were summarized using means and standard deviations. A two-sided *p* value less than 0.05 was considered statistically significant. Logistic regression models were constructed to evaluate the relationship between serum folate levels and MASLD risk. Four logistic regression models were formulated, incorporating different combinations of covariates. The Crude model did not include any adjustments for variables. Model 1 was adjusted for age, sex, race, education level, and economic status. Model 2 was further adjusted for total cholesterol (TC), HDL-C, BMI, Hypertension, Diabetes, and cardiovascular disease (CVD). Model 3, a fully adjusted model, encompassed the additional adjustments of smoking status, physical activity status, dietary energy intake, protein intake, folate intake, and alcohol intake. This logistic regression analysis categorized serum folate into tertiles, with Tertile 1 as the reference group.

### 2.2 Mendelian randomization study

#### 2.2.1 Overview

**Figure 2:**
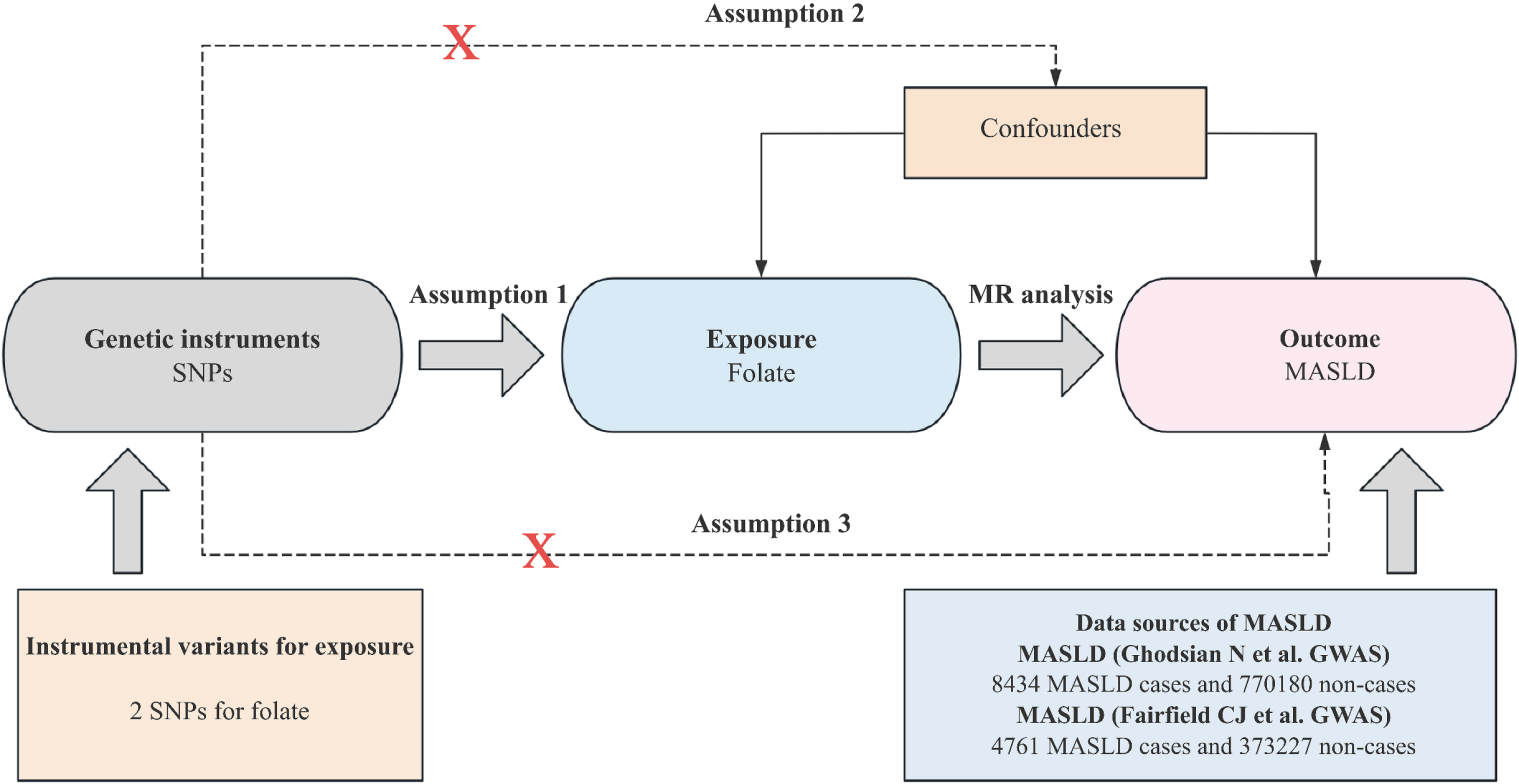
Overview of the Mendelian randomization design.

Mendelian randomization analysis hinges on three core assumptions for establishing causal inference: (1) the IV must be strongly associated with the exposure; (2) the IV should be independent of potential confounding factors; and (3) IV should affect the outcome only through exposure. Figure 2 depicts an overview of the MR design implemented in this study. Notably, all studies incorporated in the GWASs received approval from the respective review boards, thus eliminating the need for additional ethical approval or participant consent.

#### 2.2.2 Data sources

Our study utilized summary-level data derived from GWASs of European ancestry, as outlined in Table 1. Specifically, we identified single-nucleotide polymorphisms (SNPs) associated with serum folate concentrations, employing a genome-wide meta-analysis encompassing 37,645 participants of European descent. These SNPs were selected based on their significance below the genome-wide threshold (*p* < 5 × 10^−8^) and their independence from linkage disequilibrium (*r*^2^ < 0.01) (12, 20, 21). Notably, the variance in folate concentrations explained by the selected SNPs was estimated to be approximately 1.0%. Additionally, we extracted abstract-level data on MASLD from various GWAS meta-analyses, including the Electronic Medical Records and Genomics, United Kingdom Biobank, FinnGen, and Estonian Biobank studies, encompassing 8,434 MASLD cases and 770,180 controls (22). Furthermore, we included data from another GWAS comprising 4,761 MASLD cases and 373,227 controls (23).

**Table 1:** Data sources for used exposure and outcome.

Source	First author	Participants	PMID
Exposure			
Folate	Grarup N	37,465 individuals of European ancestry	23,754,956
Outcomes			
MASLD	Ghodsian N	eMERGE: 1,106 cases and 8,571 non-cases of European ancestry	34,841,290
		UK Biobank: 2,558 cases and 395,241 non-cases of European ancestry	
		Estonian Biobank: 4,119 cases and 190,120 non-cases of European ancestry	
		FinnGen: 651 cases and 176,248 non-cases of European ancestry	
	Fairfield CJ	4,761 cases and 373,227 non-cases of European ancestry	34,535,985

#### 2.2.3 MR estimates

For the primary MR analysis, IVW was employed to derive causal estimates through a meta-analysis of Wald ratio estimates for each SNP. Cochran’s *Q* statistic was utilized to assess the heterogeneity of SNP estimates across the MR associations. Additionally, the *F*-statistic was calculated, with an *F* value greater than 10 indicating a good strength of the genetic instrument (24). All MR analysis were conducted using the “TwosampleMR” program package, and odds ratios (ORs) with corresponding 95% confidence intervals (CIs) were computed. A two-sided *p* < 0.05 was considered statistically significant.

## 3 Results

### 3.1 Cross-sectional study

#### 3.1.1 Baseline characteristics of the study population based on serum folate quartile groups

The study utilized data from the National Health and Nutrition Examination Survey collected between 2017 and 2020, including 1,117 participants. Table 2 displays the baseline characteristics of the study population stratified by serum folate quartiles. Participants in the Tertile 4 group, who had the highest folate levels, were significantly older, more likely to be non-Hispanic White, had higher household incomes, had never smoked, and exhibited higher levels of physical activity compared to those in the Tertile 1 group, who had the lowest folate levels. Additionally, those in the Tertile 4 group had higher dietary folate intake, as well as elevated levels of alanine aminotransferase (ALT), aspartate transaminase (AST), and HDL-C. They also had lower waist circumference, BMI, platelet count, Fibrosis 4 index (FIB-4), and a lower prevalence of MASLD. However, no statistically significant differences were observed among the quartiles regarding TG, TC, γ-glutamyl transpeptidase (GGT), dietary energy and protein intake, alcohol consumption, education level, sex distribution, or the prevalence of diabetes, hypertension, and CVD.

**Table 2:** Baseline characteristics of the study population based on serum folate quartile groups.

Characteristics		Serum folate level				
	Overall (*n* = 1,117)	Tertile 1 (*n* = 279)	Tertile 2 (*n* = 279)	Tertile 3 (*n* = 279)	Tertile 4 (*n* = 280)	*p*
Sex, *n* (%)						0.605
Male	415 (37.2)	105 (37.6)	103 (36.9)	111 (39.8)	96 (34.3)	
Female	702 (62.8)	174 (62.4)	176 (63.1)	168 (60.2)	184 (65.7)	
Age (year), Median (IQR)	45.00(33.00, 61.00)	42.00 (31.00, 55.00)	42.00 (31.00, 56.00)	45.00 (32.00, 61.00)	56.00 (37.00, 67.00)	<0.001
Race, *n* (%)						<0.001
Mexican American	159 (14.2)	36 (12.9)	49 (17.6)	39 (14.0)	35 (12.5)	
Other Hispanic	93 (8.3)	13 (4.7)	25 (9.0)	26 (9.3)	29 (10.4)	
Non-Hispanic White	425 (38.0)	86 (30.8)	86 (30.8)	108 (38.7)	145 (51.8)	
Non-Hispanic Black	286 (25.6)	107 (38.4)	77 (27.6)	63 (22.6)	39 (13.9)	
Other race-Including Multi-racial	154 (13.8)	37 (13.3)	42 (15.1)	43 (15.4)	32 (11.4)	
Education level, *n* (%)						0.135
≤ High school	421 (37.7)	112 (40.1)	117 (41.9)	97 (34.8)	95 (33.9)	
>High school	696 (62.3)	167 (59.9)	162 (58.1)	182 (65.2)	185 (66.1)	
Economic status, *n* (%)						0.002
Low	198 (17.7)	56 (20.1)	66 (23.7)	43 (15.4)	33 (11.8)	
Middle	612 (54.8)	158 (56.6)	147 (52.7)	154 (55.2)	153 (54.6)	
High	307 (27.5)	65 (23.3)	66 (23.7)	82 (29.4)	94 (33.6)	
Smoking status, *n* (%)						<0.001
Former	275 (24.6)	58 (20.8)	63 (22.6)	73 (26.2)	81 (28.9)	
Current	183 (16.4)	71 (25.4)	49 (17.6)	35 (12.5)	28 (10.0)	
Never	659 (59.0)	150 (53.8)	167 (59.9)	171 (61.3)	171 (61.1)	
Drinking status, *n* (%)						0.254
Yes	132 (11.8)	41 (14.7)	34 (12.2)	26 (9.3)	31 (11.1)	
No	985 (88.2)	238 (85.3)	245 (87.8)	253 (90.7)	249 (88.9)	
Physical activity, *n* (%)						0.019
Yes	571 (51.1)	122 (43.7)	141 (50.5)	151 (54.1)	157 (56.1)	
No	546 (48.9)	157 (56.3)	138 (49.5)	128 (45.9)	123 (43.9)	
Diabetes, *n* (%)						0.61
Yes	217 (19.4)	51 (18.3)	51 (18.3)	53 (19.0)	62 (22.1)	
No	900 (80.6)	228 (81.7)	228 (81.7)	226 (81.0)	218 (77.9)	
Hypertension, *n* (%)						0.68
Yes	453 (40.6)	120 (43.0)	106 (38.0)	112 (40.1)	115 (41.1)	
No	664 (59.4)	159 (57.0)	173 (62.0)	167 (59.9)	165 (58.9)	
CVD, *n* (%)						
Yes	115 (10.3)	31 (11.1)	30 (10.8)	23 (8.2)	31 (11.1)	0.633
No	1,002 (89.7)	248 (88.9)	249 (89.2)	256 (91.8)	249 (88.9)	
Anthropometric measurements						
Waist circumference (cm), Median (IQR)	100.00 (88.50, 112.40)	103.30(91.65, 117.45)	102.00 (90.50, 114.50)	97.10 (85.90, 109.70)	97.60 (86.20, 108.75)	<0.001
BMI, *n* (%)						<0.001
Under/normal weight	273 (24.4)	48 (17.2)	59 (21.1)	78 (28.0)	88 (31.4)	
Overweight	316 (28.3)	63 (22.6)	84 (30.1)	83 (29.7)	86 (30.7)	
Obesity	528 (47.3)	168 (60.2)	136 (48.7)	118 (42.3)	106 (37.9)	
Dietary characteristics						
TKCAL (kcal), Median (IQR)	1845.50 (1399.50, 2345.50)	1844.00 (1356.25, 2411.00)	1800.00 (1402.25, 2336.75)	1908.50 (1441.50, 2427.25)	1813.50 (1413.00, 2231.88)	0.489
TPROT (mg), Median (IQR)	69.32 (53.03, 91.14)	66.36 (48.36, 91.64)	70.00 (53.91, 91.13)	73.50 (54.86, 94.16)	67.76 (53.80, 86.71)	0.103
TFOLA (mcg), Median (IQR)	304.50 (218.50, 409.50)	266.50 (192.00, 360.50)	297.50 (215.25, 396.75)	316.50 (226.25, 436.50)	338.75 (256.25, 447.00)	<0.001
Biochemical indicators						
ALT (μ/L), Median (IQR)	17.00 (12.00, 24.00)	15.00 (11.00, 21.00)	17.00 (12.00, 25.00)	17.00 (12.00, 25.50)	17.00 (13.00, 23.00)	0.002
AST (μ/L), Median (IQR)	18.00 (15.00, 22.00)	16.00 (14.00, 20.75)	18.00 (15.00, 23.00)	18.00 (15.00, 23.00)	19.00 (16.00, 23.00)	<0.001
GGT (IU/L), Median (IQR)	19.00 (14.00, 29.00)	20.00 (13.00, 27.50)	21.00 (14.50, 30.00)	20.00 (13.00, 29.00)	19.00 (14.00, 27.00)	0.243
TG (mg/dL), Median (IQR)	101.00 (72.00,147.00)	96.00 (70.50, 138.00)	106.00 (78.00, 146.50)	96.00 (67.00, 149.00)	108.00 (74.00, 151.00)	0.093
HDL-C (mg/dL), Median (IQR)	52.00 (43.00, 61.00)	49.00 (42.00, 58.00)	51.00 (43.00, 60.00)	52.00 (43.00, 63.00)	54.00 (43.00, 64.00)	0.003
TC (mg/dL), Median (IQR)	178.00 (155.00, 206.00)	177.00 (153.00, 204.00)	182.00 (155.00, 207.00)	175.00 (156.00, 205.00)	180.50 (158.75, 205.25)	0.43
PLT, Median (IQR)	246.00 (203.00, 284.00)	256.00 (214.50, 299.50)	250.00 (198.00, 283.00)	239.00 (207.50, 282.00)	236.00 (199.00, 272.25)	<0.001
FIB-4	0.80 (0.53, 1.23)	1.02 (0.63, 1.48)	0.71 (0.50, 1.19)	0.85 (0.57, 1.17)	0.67 (0.46, 1.03)	<0.001
MASLD, *n* (%)						0.002
Yes	547 (49.0)	159 (57.0)	145 (52.0)	122 (43.7)	121 (43.2)	
No	570 (51.0)	120 (43.0)	134 (48.0)	157 (56.3)	159 (56.8)	

CVD, Cardiovascular disease; BMI, Body mass index; TKCAL, Dietary energy intake; TPROT, Dietary protein intake; TFOLA, Dietary folate intake; ALT, Alanine aminotransferase; AST, Aspartate transaminase; GGT, γ-Glutamyl transpeptidase; TG, Triglyceride; HDL-C, High-density lipoprotein cholesterol; TC, Total cholesterol; FIB-4, Fibrosis 4 index; MASLD, Metabolic dysfunction-associated steatotic liver disease.

#### 3.1.2 Associations between MASLD and serum folate (all participants)

Table 3 presents the crude and adjusted odds ratios for the association between serum folate levels and MASLD. Our results indicate a significant inverse relationship between serum folate levels and MASLD in all models. When compared to the crude model, Model 1 exhibited a modest increase in the strength of this association after adjusting for confounders such as age, sex, race, education level, and economic status (OR: 0.988, 95% CI: 0.982–0.994, *p* < 0.001). Model 2, which builds upon Model 1, incorporates additional adjustments for TC, HDL-C, BMI, Hypertension, Diabetes status, and CVD (OR: 0.991, 95% CI: 0.985–0.998, *p* = 0.011). Finally, Model 3 extends Model 2 by incorporating further adjustments for smoking status, physical activity status, TKCAL, TPROT, TFOLA, and drinking status. Notably, the association remained stable in Model 3 (OR = 0.991, 95% CI: 0.984 ~ 0.998, *p* = 0.017).

**Table 3:** Association between serum folate levels and MASLD (all participants), NHANES 2017–2020.

Models	MASLD OR (95%CI)	*p* value
Crude Model	0.991(0.986 ~ 0.997)	0.003
Model 1	0.988(0.982 ~ 0.994)	<0.001
Model 2	0.991(0.985 ~ 0.998)	0.011
Model 3	0.991(0.984 ~ 0.998)	0.017

Crude model, not adjusted; Model 1, adjusted for age, sex, race, education level, and economic status; Model 2, Model 1 + TC, HDL-C, BMI, hypertension status, diabetes status, and CVD; Model 3, Model 2 + smoking status, physical activity status, TKCAL, TPROT, TFOLA, and drinking status; OR, Odds ratio.

#### 3.1.3 Association between MASLD and serum folate levels (tertiles)

Table 4 depicts the association between varying concentrations of serum folate and the risk of developing MASLD. Logistic regression analysis revealed that individuals in the Tertile 3 group (≥48.6 nmol/L) exhibited a lower risk of MASLD compared to those in the Tertile 1 group (< 22.3 nmol/L). In the crude model, which did not account for covariates, the risk of MASLD in the Tertile 3 group was reduced by 42% (OR: 0.58, 95% CI: 0.42–0.82, *p* = 0.002). A similar risk reduction was observed in the Tertile 2 serum folate group. Upon adjusting for covariates, the protective effect of higher serum folate concentrations on the risk of MASLD in the Tertile 3 group remained stable across Models 1–3 (Model 1: OR: 0.50, 95% CI: 0.35–0.71, *p* < 0.001; Model 2: OR: 0.58, 95% CI: 0.39–0.87, *p* = 0.008; Model 3: OR: 0.58, 95% CI: 0.38–0.88, *p* = 0.010). Notably, the risk of MASLD in Model 3 was slightly reduced compared to Model 1.

**Table 4:** Association between serum folate levels and MASLD (Tertiles), NHANES 2017–2020.

Folate (nmol/L)	MASLD							
	Crude model		Model 1		Model 2		Model 3	
	OR (95%CI)	*p*	OR (95%CI)	*p*	OR (95%CI)	*p*	OR (95%CI)	*p*
Tertiles								
Tertile1	1.00 (Ref)		1.00 (Ref)		1.00 (Ref)		1.00 (Ref)	
Tertile2	0.70 (0.52, 0.93)	0.015	0.67(0.50,0.90)	0.007	0.72(0.51,0.10)	0.047	0.70(0.50,0.99)	0.042
Tertile3	0.58 (0.42, 0.82)	0.002	0.50(0.35,0.71)	<0.001	0.58(0.39,0.87)	0.008	0.58(0.38,0.88)	0.010
*p* for trend	0.76 (0.65, 0.90)	0.001	0.71(0.59,0.84)	<0.001	0.76(0.62,0.93)	0.008	0.76(0.62,0.94)	0.010

Crude model, not adjusted; Model 1, adjusted for age, sex, race, education level, and economic status; Model 2, Model 1 + TC, HDL-C, BMI, hypertension status, diabetes status, and CVD; Model 3, Model 2 + smoking status, physical activity status, TKCAL, TPROT, TFOLA, and drinking status; OR, odds ratio.

### 3.2 Mendelian randomization study

**Figure 3:**
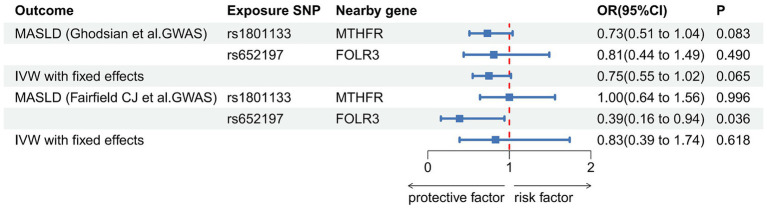
Association of genetically predicted concentrations of serum folate and risk of metabolic dysfunction-associated steatotic liver disease. The estimates for folate are derived from the IVW model.

Utilizing the SNP selection criteria, Ghodsian et al. and Fairfield et al. were left with only two SNPs each to analyze the association between circulating folate levels and MASLD. The Data sources and MR estimate describe the associated exclusion SNPs. Considering the *F*-statistics of the selected SNPs, which range from 39.35 to 144 (Table 5), our findings are unlikely to be influenced by weak instruments. In both the primary analysis (OR: 0.75, 95% CI: 0.55–1.02, *p* = 0.065) and the secondary analysis (OR: 0.83, 95% CI: 0.39–1.74, *p* = 0.618) using the IVW model, the association failed to reach the significance threshold of *p* < 0.05, indicating that the observed association is not causal (Table 6; Figure 3).

**Table 5:** Summary information for SNPs used as genetic instruments for Mendelian randomization analyses.

Folate concentration								
SNP	Nearby gene	EA	NEA	EAF	β	SE	*p* value	*F* statistic
rs1801133	MTHFR	G	A	0.67	0.096	0.008	9.50e−53	144
rs652197	FOLR3	C	T	0.18	0.069	0.011	1.40e−12	39.35

SNP, Single-nucleotide polymorphism; EA, Effect allele; OEA, Other effect allele; SE, Standard error; EAF, Effect allele frequency.

**Table 6:** Associations of serum folate and risk of MASLD in single dataset.

Outcome	Exposure	*p* value	OR (95%CI)	*p* value for Cochran’s *Q* test	Inverse-variance weighted	
					95%CI	*p* value
MASLD	rs1801133	0.083	0.73 (0.51, 1.04)	0.779	0.75 (0.55,1.02)	0.065
(Ghodsian N. et al. GWAS)	rs652197	0.490	0.81 (0.44, 1.49)			
MASLD	rs1801133	0.996	1.00 (0.64, 1.56)	0.061	0.83 (0.39,1.74)	0.618
(Fairfield C. J. et al. GWAS)	rs652197	0.036	0.39 (0.16, 0.94)			

## 4 Discussion

In this study, we integrated a cross-sectional survey utilizing data from the NHANES spanning 2017–2020 with a two-sample MR analysis of summary GWAS data to explore the relationship between serum folate levels and the risk of MASLD. In observational analyses, we observed an inverse association between higher serum folate concentrations and a reduced risk of MASLD. This finding remained robust even after adjusting for various potential confounders, such as sociodemographic characteristics, lifestyle factors, hypertension, and diabetes. In the MR analysis, two separate GWAS datasets of MASLD generated similar results, but the association failed to reach the significance threshold of *p* < 0.05, suggesting that the observed association is not causal. Our results indicate that elevated folate levels may be protective against MASLD, though further investigation is warranted to confirm this hypothesis.

The relationship between folate and MASLD has garnered significant attention in previous research, offering valuable insights. Notably, a randomized controlled trial investigating the effects of a green Mediterranean diet on intrahepatic fat revealed that subjects experiencing the most significant increase in serum folate exhibited greater reductions in intrahepatic fat (IHF), suggesting the efficacy of serum folate in mitigating MASLD risk (25). Additionally, the study by Mahamid et al. identified a significant correlation between lower folate levels and the severity of liver fibrosis (26). A recent meta-analysis encompassing data from 12 studies corroborated these findings and reinforced the inverse association between serum folate concentration and MASLD risk through primary and subgroup analyses (12). These concordant observations, consistent with the cross-sectional analysis presented in this study, underscore the potential role of folate in the prevention and management of MASLD.

However, some studies have reached contrasting conclusions. A meta-analysis encompassing eight studies published up to 2015 found no significant difference in folate concentrations between patients with and without MASLD (13). Li et al. (15), utilizing data from NHANES 1999–2004, suggested no direct association between serum folate and MASLD. Similarly, Liu et al. (14) examined the relationship between vitamins and MASLD but did not observe an association between dietary folate intake and MASLD. This discrepancy may stem from variations in study years, sample sizes, covariate adjustments, dietary intake measurements, and serum folate levels. Furthermore, our MR analysis revealed no significant trend in the association between genetically predicted serum folate concentration and MASLD risk. Therefore, more extensive GWAS are required to enhance our understanding of micronutrient regulation and optimize the instrumental variables in MR analysis. Additionally, clinical studies with larger sample sizes are necessary to clarify the relationship between serum folate and MASLD.

The inverse association between serum folate levels and MASLD is supported by multiple mechanisms. Firstly, folate deficiency decreases methylation capacity, which affects carnitine and phosphatidylcholine (PC) synthesis, promoting MASLD progression (27). Secondly, folic acid exhibits potent antioxidant properties, enhancing mitochondrial β-oxidation, reducing oxidative stress, and inhibiting peroxisome proliferator-activated receptor γ (PPARγ), a key regulator of lipogenesis and hepatic TG accumulation, thereby mitigating hepatic steatosis (28). Thirdly, elevated serum folate levels attenuate the expression of proinflammatory cytokines (IL-6 and TNF-α), inhibiting Kupffer cell recruitment and activation, ultimately reducing the risk of MASLD (29); Fourthly, folic acid modulates liver microRNAs, contributing to lower blood glucose and lipid concentrations, improved insulin sensitivity, and enhanced liver function (30). Fifthly, folate deficiency disrupts the fibroblast growth factor (FGF) pathway and activates adipokines and inflammatory factors, thereby fostering fatty liver disease development (31, 32). Furthermore, Kim et al. (33) demonstrated that folic acid supplementation activates the liver kinase B (LKB1)/AMP-activated protein kinase (AMPK)/acetyl coenzyme A carboxylase (ACC) pathway, increases hepatic S-adenosylmethionine, and inhibits hepatic lipogenesis, ultimately improving hepatic steatosis.

The current study boasts several notable strengths. Firstly, the utilization of a large cross-sectional study based on NHANES, alongside the integration of two-sample MR analysis, provides a robust framework for exploring the relationship between serum folate concentration and the risk of MASLD. The cross-sectional approach allows for an epidemiological evaluation of this relationship, while the MR studies effectively mitigate potential confounders and reverse causality. By independently examining two associations and then combining the data, we increased case numbers, thereby enhancing the power of our established associations compared to previous MR studies. However, some limitations need to be acknowledged. Firstly, despite our efforts to include a comprehensive set of covariates in the cross-sectional study to minimize bias from potential confounders, residual confounding may persist due to the possibility of unaccounted variables. Secondly, in this study, we used non-invasive VCTE to determine whether subjects had liver steatosis and liver fibrosis instead of using liver biopsy, which inevitably leads to bias in the diagnosis of MASLD. Thirdly, we could not perform a sensitivity analysis for folate due to the use of less than three SNPs in the analysis. However, it is worth noting that the association for folate was derived from an SNP located within the gene encoding methylenetetrahydrofolate reductase (MTHFR), indicating that this association is unlikely to be biased by pleiotropy from a biological perspective. Fourthly, MR relies on the assumption of a linear relationship between exposure and outcome, which may only sometimes hold in the presence of nonlinear associations. Fifthly, MR considers the lifelong impact of micronutrient status by utilizing genetic variation as an instrumental variable. Therefore, our MR results should not be generalized to extreme cases of micronutrient status. Lastly, it is essential to note that our cross-sectional and genetic data were obtained from different samples, with the MR Study focusing on individuals of European ancestry. In contrast, the cross-sectional study encompassed a multi-racial United States population. This may introduce some heterogeneity in our results, which should be considered when interpreting our findings.

## 5 Conclusion

In conclusion, despite cross-sectional studies indicating a significant association, our study does not endorse a causal relationship between genetically determined serum folate levels and the risk of MASLD. However, observational study results may be skewed due to uncontrolled confounding factors. Consequently, based on the current research, there is insufficient evidence to suggest that elevated serum folate levels may contribute to preventing or treating MASLD. While our findings are promising, further research with larger sample sizes is warranted to strengthen the validation of our observations and ensure broader applicability.

## Data Availability

The raw data supporting the conclusions of this article will be made available by the authors, without undue reservation.
